# Effects of Probiotic-Fermented Deer Bone Water Extract on Immune Regulation and Gut Microbiota in Rheumatoid Arthritis via the NOTCH Signaling Pathway

**DOI:** 10.3390/foods14213802

**Published:** 2025-11-06

**Authors:** Junxia Ma, Yingshan Jiang, Yue Teng, Ting Ren, Yanchao Xing, Aoyun Li, Zhongmei He, Weijia Chen, Ying Zong, Rui Du

**Affiliations:** 1 College of Chinese Medicinal Material, Jilin Agricultural University, Changchun 130118, China; jxiama@163.com (J.M.); 15754307745@163.com (Y.J.); 15065646082@163.com (Y.T.); r15834417002@163.com (T.R.); 18345780732@163.com (Y.X.); 18935289181@163.com (A.L.); heather78@126.com (Z.H.); chenweijia_jlau@163.com (W.C.); 2 Laboratory of Production and Product Application of Sika Deer of Jilin Province, Jilin Agricultural University, Changchun 130118, China; 3 College of Agriculture, Yanbian University, Yanji 133002, China

**Keywords:** deer bones, rheumatoid arthritis, fermentation, transcriptomics, gut microbiota, short-chain fatty acids

## Abstract

Rheumatoid arthritis (RA) is a chronic autoimmune inflammatory disease, and current treatments are limited by significant side effects. Deer bone, which is rich in proteins and various active compounds, possesses anti-inflammatory and bone-health-promoting properties. However, its fermented product’s effects on RA treatment remain unexplored. In this study, we evaluated the therapeutic effects of probiotic-fermented deer bone aqueous extract (BbF) in an adjuvant arthritis (AA) rat model, combined with LPS-stimulated RAW264.7 macrophage models. In vivo experiments showed that BbF significantly reduced paw swelling, arthritis index, and improved bone mineral density. BbF also alleviated synovial hyperplasia and inflammatory cell infiltration. It suppressed pro-inflammatory cytokines (TNF-α, IL-1β, etc.) and inhibited macrophage migration and invasion. Transcriptomic analysis revealed significant enrichment of the Notch signaling pathway, and Western blot confirmed the downregulation of Notch3, Notch4, DLL4, and Jagged1 proteins. BbF also restored gut microbiota homeostasis, increasing beneficial bacteria such as *Firmicutes* and *Clostridia*, while decreasing potential pathogens like *Proteobacteria*, *Gammaproteobacteria*, and *Escherichia-Shigella*. Furthermore, BbF enhanced short-chain fatty acids (SCFCs) production, including butyrate and caproic acid. These results indicate that BbF alleviates RA by inhibiting the Notch signaling pathway and regulating gut microbiota, providing new insights for the development of functional foods with immune-modulatory properties.

## 1. Introduction

Rheumatoid arthritis (RA) is a chronic autoimmune illness that is difficult to manage and affects 0.5–1% of people worldwide [1]. The etiology of the condition is intricate and multi-dimensional [2]. It is hypothesized that the condition arises from interactions among multiple factors, including genetic susceptibility, environmental triggers, immune dysregulation, and metabolic dysfunction—encompassing both systemic metabolic disturbances (e.g., insulin resistance, dyslipidemia, and sarcopenia) and cellular metabolic reprogramming (e.g., aberrant glycolysis and imbalanced lipid/amino acid metabolism in immune and synovial cells) [3]. The pathological characteristics of RA are distinct and severe, and are mainly manifested as persistent inflammation of the synovial membrane of the joint, progressive erosion of cartilage, and destruction of bone. These pathological processes eventually lead to changes in the joint structure, which greatly reduces the quality of life of patients [4]. Presently, no complete cure for RA is available [5]. The primary focus of clinical treatment is drug intervention. The administration of medication over an extended period may result in adverse effects, including gastrointestinal damage and an elevated susceptibility to infection [6]. However, in the quest to better manage chronic diseases and improve outcomes in RA, there is growing interest in the role of dietary and nutritional intake. Protein consumption plays a complex and critical role in the management of RA. It serves not only as a fundamental nutrient for maintaining overall muscle mass and immune function but may also influence inflammatory responses through multiple pathways [7]. It has been shown that NOTCH signaling has been widely studied in different chronic inflammatory diseases (such as RA, Inflammatory bowel disease and Systemic Lupus Erythematosus) [8,9]. In RA, the genes and ligands related to NOTCH signaling are highly expressed [10], and it is generally believed that inhibition of NOTCH signaling benefits the treatment of RA. (DAPT is a common γ-secretase inhibitor which effectively blocks the NOTCH signaling pathway.) [11]. The aim of this study is to investigate whether the ameliorative effects of BbF on RA are achieved through inhibition of the NOTCH signaling pathway.

Deer bones, derived from the bones of precious sika deer (*Cervus nippon* Temminck) or red deer (*Cervus elaphus* Linnaeus). Deer bones are characterized by a high protein content, in addition to various minerals and trace elements. Their properties have been described as follows: “tonifying deficiency and strengthening muscles and bones” [12]. It has been demonstrated that these components have a beneficial effect on bone health, by virtue of their ability to reduce inflammatory responses and enhance immunity [13]. Research has demonstrated that deer bone extract has been shown to assist in the alleviation of osteoarthritis by repairing cartilage damage caused by sodium iodoacetate (MIA) in models of OA [14,15]. However, no studies have reported the mechanism and effect of probiotic-fermented deer bone aqueous extract (BbF) on the remission and treatment of RA.

The microbiota plays a crucial role in maintaining physiological homeostasis in the human body [16]. Some studies have confirmed that the human gut harbors numerous bacterial communities that together form a complex ecosystem of gut microbiota [17], which contribute significantly to endocrine function through the secretion of metabolites that interact with the host’s endocrine system [18,19]. An imbalance in the gut microbiota disrupts the host’s immune homeostasis, leading to abnormal regulation of the immune system, which manifests itself in a disturbed immune response, a sustained inflammatory response, and associated pathologic processes. This imbalance is an important pathological basis for the development of immune-mediated diseases such as RA, and is closely related to disease progression and prevention [20].

The fermentation technology of probiotics, following continuous expansion, has been widely applied in the fields of food and medicine, which has also brought more development opportunities. It is evident that *Bifidobacterium breve* plays a pivotal role in the human intestinal tract as a probiotic [21], and it has also been shown to have a certain alleviating effect on RA. The present article aims to utilize *Bifidobacterium breve* to ferment the water extract of deer bones, with a view to further elucidating the research on BbF and the mechanism of regulating the immune response of RA and stabilizing the intestinal flora. The study provides novel concepts and a theoretical framework for the relief therapy of RA, and also establishes a scientific foundation for the development of healthier functional deer bone derivative products.

## 2. Materials and Methods

### 2.1. Materials and Reagents

Sika deer bones were purchased from Haixia Deer Products Distribution Office, Shuangyang District, Jilin Province, China. Bifidobacterium breve was purchased from BNCC Beina Chuanglian Biotechnology Co., Ltd. (Beijing, China). The ELISA kits for TNF-α, IL-1β, IL-6, IL-12, VEGF and RF-Ab were purchased from Preferred Biotechnology Co., Ltd. (Shanghai, China). DAPT (Cat. No. HY-13027) was obtained from MedChemExpress (MCE, Shanghai, China).

MTX (Cat. No. M813626) was purchased from Macklin Biochemical Co., Ltd. (Shanghai, China).

Methanol and acetonitrile (HPLC grade) were purchased from Sigma-Aldrich ((St. Louis, MO, USA). Formic acid, 3-NPH (3-nitrophenylhydrazine), EDC, and pyridine were obtained from Shanghai Aladdin Biochemical Technology Co., Ltd. (Shanghai, China). All reference standards were also sourced from Shanghai Aladdin Biochemical Technology Co., Ltd.

### 2.2. Preparation of Fermentation Products

Deer leg bones were defatted, decalcified, and subsequently extracted with hot water (95 °C, 1:10 ratio, thrice). The combined extracts were concentrated and lyophilized (Christ 21529) to obtain a fully water-soluble deer bone extract powder. Fermentation was carried out in a 2% deer bone water extract medium, which was supplemented with 0.5% glucose and 0.5% yeast extract as auxiliary carbon and nitrogen sources. The fermentation process was optimized using inoculum size, temperature, and time as key parameters to evaluate its effect on the protein content of deer bone water extract, with the optimum conditions being 6% inoculum of *Bifidobacterium breve*, 37 °C, and 45 h. After fermentation, the product was centrifuged at 3000 rpm for 15 min, 4 °C to remove *Bifidobacterium brev*. The supernatant was then filtration (0.22 μm filter), concentrated and lyophilized to obtain the target product (BbF) as a powder, The total protein concentration was determined using a BCA assay kit (Beyotime Biotechnology, Shanghai, China) and the process was conducted in triplicate for validation. the obtained BbF demonstrated good stability.

### 2.3. Measurement of Protein Composition by Label-Free Proteomics

#### 2.3.1. LC-MS/MS Analysis

Peptides were desalted using a C18 Cartridge, lyophilized, and then reconstituted in 40 μL of 0.1% formic acid (FA) solution. Peptide quantification was performed by measuring the absorbance at OD280.

Separation of each sample was performed using a nanoflow HPLC system. The mobile phases consisted of 0.1% formic acid in water (Buffer A) and 0.08% formic acid in 80% acetonitrile (Buffer B). The column was equilibrated with 100% Buffer A and load the sample into a precolumn of mass spectrometry (C18 3 μm 100 μm × 20 mm, Thermo Scientific, Waltham, MA, USA) before separating it with an analytical column (C18 1.9 μm 150 μm × 120 mm, Thermo Scientific).

The samples were separated using capillary HPLC and analyzed using an Q Exactive HF-X mass spectrometer (Thermo Scientific, Waltham, MA, USA). Parameter setting: analyzing time 88 min, detecting method of positive ion, scanning range of parent ion is 300~2000 *m*/*z*, primary mass spectrogram is 120,000, AGC target is 3 × 10^6^, primary maximum IT is 20 ms, and Doppler exclusion time is 15.0 s. Polypeptide and its mass-to-charge ratio are obtained according to the following method: MS2 Activation Type is HCD, Isolation window is 1.6 *m*/*z*, Microscans is 1, Secondary Maximum IT is 30 ms, and Normalized Collision Energy is 27 eV [22].

#### 2.3.2. Quantitative Analysis of Differential Proteins, GO Function Annotation, and KEGG Pathway Annotation

Using EBI database resources and InterProScan (5.52-86.0) software, we performed GO functional annotation on all differentially expressed proteins. In addition, the number of differentially expressed proteins was counted at the GO secondary functional annotation level. KEGG, as a key public database in the field of Pathway, identifies the core biochemical and metabolic pathways and signaling pathways in which proteins are involved by means of Pathway analysis.

### 2.4. Animal Experiment and Modeling

Healthy female Wistar rats (4–8 weeks old, 180–220 g, SPF grade) were acquired from Easylabs Laboratory Animal Science and Technology Co. Ltd. (Changchun, China), under license No. SCXK (JI) 2023-0002, all animal tests were approved by Jilin Agricultural University’s Animal Welfare and Ethics Committee (Ethical Review Approval No. 20,211,011,003, 11 October 2021).and kept in a setting with unrestricted access to food and water. After a one-week acclimation period, rats were randomly divided into 6 groups. Adjuvant-induced arthritis (AA) was induced in rats [23]. For the model (AA) group, a microsyringe was used to subcutaneously inject complete Freund’s adjuvant (CFA 0.1 mL) into the metatarsal region of the left hind paw in all groups. The control group (CK) was injected with an equal volume (0.1 mL) of sterile saline at the same site. 7 days post-induction, the AA group received a booster injection of 0.1 mL of incomplete Freund’s adjuvant (IFA) at the same site, while the CK group was administered 0.1 mL of sterile saline. Methotrexate (MTX) (Cat. No. M813626) was purchased from Macklin Biochemical Co., Ltd. (Shanghai, China) was used as the positive control, and DAPT ((Cat. No. HY-13027) was obtained from MedChemExpress (MCE, Monmouth Junction, NJ, USA)) was employed as a γ-secretase inhibitor. For the purpose of administering the medicine, the rats were thereafter randomly assigned to 6 groups (n = 6): CK group (2 mL sterile saline), AA group, MTX group (0.5 mg/kg), DAPT group (100 ng/kg), BbFH group (800 mg/kg), and BbFL group (200 mg/kg). CK group and AA group were gavaged with an equivalent volume of saline based on body weight, while the remaining groups received their respective treatments by gavage according to the designated dosing regimens. The degree of joint swelling was observed in each group. We euthanized the rats 24 h after the final treatment of the 21-day course by intraperitoneal injection of sodium pentobarbital (200 mg/kg). We then collected the synovial tissues and knee joints, which were stored at −80 °C for future analysis. Additionally, the spleen and thymus were harvested and weighed to calculate the spleen and thymus indices.

### 2.5. Changes in Toe Volume, Arthritisl Score and Body Weight

The degree of ftoe volume in each group of rats was measured and recorded using a paw volume plethysmometer. A fixed mark was placed on the left ankle joint of each rat, and the ankle was extended to align the mark with the water surface of the plethysmometer. The instrument readings were then documented. On days 0, 7, 14, 21, 28, 35, and 42 post-modeling, the extent of toe volume, arthritisl score, and body weight for each group of rats were assessed and recorded. (arthritisl score: Grade 0: indicates normal or no inflammation; Grade 1: Slight redness and swelling of the ankle. Grade 2: Slight redness and swelling of the tarsus or ankle, single area. Grade 3: Moderate redness and swelling of the ankle extending to the metatarsal area. Grade 4: Severe erythema, joint deformity with stiffness, area of erythema expanding again

### 2.6. Micro-Computed Tomography

A high-resolution micro-computed tomography (micro-CT) scanner (Scanco Medical VivaCT 80, Brüttisellen, Switzerland) was performed on all rats to evaluate the joint spaces and the degree of bone erosion, and analyzed the parameters bone volume (BV/TV), bone mineral density (BMD), trabecular number (Tb.N) and trabecular thickness (Tb.Th).

### 2.7. Histological Hematoxylin and Eosin (HE) Staining Analysis

At the end of the experiment, all rats were euthanized by an intraperitoneal overdose of sodium pentobarbital (200 mg/kg). Death was confirmed by the absence of a pupillary reflex and cessation of heartbeat prior to tissue collection. The rats had their knees removed, and care was taken to remove the soft tissue while preserving the capsule. The joint tissues were fixed for 24 h in 10% neutral buffered formalin (about 10 times the volume of the joint), HE staining was used for histological analysis of bone tissues from experimental rats. The bones were excised, rinsed with saline, dehydrated, and embedded in paraffin. Tissue sections were deparaffinized and stained with HE, then examined under a light microscope (NIKON Eclipse ci, Tokyo, Japan) to observe the morphological changes in bone tissue.

### 2.8. Western Blot Analysis

Western blotting was used to detect the expression of specific proteins in bone tissue, including NOTCH3, NOTCH4, DLL4, and Jagged1 and the internal control β-actin. Total proteins were extracted from bone tissues using RIPA buffer. Equal amounts of protein were separated by SDS-PAGE and transferred to PVDF membranes. Primary antibody incubation was carried out overnight at 4 °C. After binding with the secondary antibody Protein bands were visualized using the ECL chemiluminescence system.

### 2.9. Multi-Omics Sequencing and Bioinformatics Analysis

#### 2.9.1. Transcriptome Sequencing and Enrichment Analysis

Total RNA was extracted from bone tissues of experimental rats and used for transcriptome sequencing. RNA quality and concentration were assessed using a NanoDrop spectrophotometer and agarose gel electrophoresis. The RNA was used for library construction with the NEBNext^®^ Ultra™ RNA Library Prep Kit for Illumina^®^ (NEB, Ipswich, MA, USA), followed by sequencing on the Illumina Novaseq platform. Differentially expressed genes (DEGs) were identified with a *p*-value < 0.05 and an absolute fold change ≥2. GO functional enrichment and KEGG pathway analysis were performed using the Metascape online database (https://metascape.org/, accessed on 21 April 2025 to elucidate the biological functions and associated metabolic pathways of the DEGs.

#### 2.9.2. 16S Ribosomal RNA Gene Sequencing

To analyze the microbiota composition, 16S rRNA gene sequencing was performed. Microbial DNA was extracted from the ileal content of rats using the QIAamp DNA Stool Mini Kit (Qiagen, Hilden, Germany). The DNA quality was assessed by agarose gel electrophoresis and quantified using a Qubit 2.0 Fluorometer (ThermoFisher Scientific, Waltham, MA, USA). The V3-V4 hypervariable regions of the 16S rRNA gene were amplified by PCR using specific primers, then purified with AMPure XP Beads (Beckman Coulter, Brea, CA, USA). Sequencing libraries were constructed and quality-checked before sequencing on the Illumina HiSeq 2500 platform according to the manufacturer’s instructions.

### 2.10. Analysis of SCFAs

Samples were thawed at 4 °C and 20 mg aliquots were processed in 2 mL centrifuge tubes. After adding 0.40 mL of 50% acetonitrile-water solution, the mixtures were vortexed for 1 min, ultrasonicated at 4 °C for 30 min, and centrifuged at 12,000 rpm (4 °C, 15 min). The supernatant was derivatized with 3-NPH (200 mM) and EDC (120 mM, containing 6% pyridine) in a 2:1:1 (*v*/*v*/*v*) ratio. Following vortex mixing and derivatization at 40 °C for 1 h, the solution was centrifuged under the same conditions, filtered through a 0.22 μm membrane, and diluted 10- and 200-fold for LC-MS/MS analysis.

Analysis was performed on a Waters Acquity UPLC system coupled to an AB SCIEX 5500 QQQ-MS with an Acquity UPLC HSS T3 column (1.8 μm, 2.1 × 100 mm). Chromatographic conditions included: column temperature 40 °C, flow rate 0.300 mL/min, mobile phase A (water with 0.05% formic acid) and B (acetonitrile with 0.05% formic acid), injection volume 4 μL, and 8 min gradient elution. Mass spectrometry parameters were: ESI ion source, ion spray voltage 4500 V, temperature 450 °C, curtain gas 35 arb, collision gas 9 arb, and ion source gases 1 and 2 at 55 arb each. Quantification used external standards with MultiQuant software (version 3.0.3; SCIEX, Framingham, MA, USA) for peak integration and calculation [24].

### 2.11. Cell Viability Assay

Macrophages (RAW264.7) (from the laboratory at Jilin Agricultural University) in the logarithmic growth phase were inoculated in 96-well plates at a density of 1 × 10^5^ cells/mL and cultured at 37 °C under 5% CO_2_ for 12 h. BbF solutions at concentrations of 15.625, 31.25, 62.5, 125, 250, 500, and 1000 μg/mL were added, and a blank control group was set up, and the incubation was continued for 24 h. Each sample was set up in three replicate wells. Three replicate wells were set up for each sample. Add 10 μL of CCK-8 and incubate at 37 °C 5% CO_2_ for 40 min, avoiding light. At the end of the incubation, the absorbance value at 450 nm was measured by an enzyme labeler, and the cell proliferation rate was calculated.

### 2.12. Evaluating Migration and Invasion in RA-RAW264.7 Cells

Cells were divided into groups for culture (CK group, LPS group, MTX group, DAPT group, BbFL group, BbFM group, and BbFH group). When cell confluence reached approximately 80%, all groups except the CK group were induced with LPS (1 μg/mL in the culture medium). The 6-well plate was incubated at 37 °C with 5% CO_2_ for 12 h. After 12 h, 3 horizontal lines were scraped in each well using a 100 μL micropipette tip. The CK group received fresh medium, while the model group was replenished with fresh medium containing 1 μg/mL LPS. and the positive drug group, the inhibitor group, and the experimental group were replaced with the corresponding concentrations of MTX, DAPT, BbFL, BbFM, and BbFH groups, respectively, photos were taken in the stationary area at 0 h. The plate was then returned to the incubator for an additional 24 h, after which images were captured again. The migration distance of cells in the scratch wound was quantified using ImageJ software (Fiji Is Just ImageJ).

Matrigel was thawed at 4 °C and diluted 1:8 with pre-chilled culture medium. Then, 60 µL of the solution was evenly coated onto the bottom of each chamber and polymerized at room temperature for 3 h. RA-RAW264.7 cells were adjusted to 1 × 10^5^ cells per 100 µL after culture, and 200 µL of serum-free cell suspension was seeded per chamber. The chambers were placed in a 24-well plate with 500 µL of complete medium (with serum) added to each outer well. Following 12 h of incubation, the medium was aspirated, and non-migrated cells on the inner surface were gently removed with a cotton swab. Subsequently, the chambers were fixed with 4% paraformaldehyde for 10 min and washed thrice with PBS. Cells were stained with 0.1% crystal violet (500 µL) for 20 min, rewashed with PBS, and any residual liquid was absorbed carefully. The number of invading RA-RAW264.7 cells was observed under a microscope and quantitatively analyzed.

### 2.13. Cytokines Assay In Vitro

Following drug treatment and culture, the levels of inflammatory cytokines (TNF-α, IL-1β, and IL-6) in the supernatant of RA-RAW264.7 cells were measured and quantitatively analyzed using commercial ELISA kits.

### 2.14. Immunofluorescence Detection

According to the grouping and drug administration scheme described in Section 2.11, after the respective treatments, cells were washed and permeabilized with 0.5% Triton X-100 for 15 min, then blocked with 10% sheep serum for 1 h. Subsequently, they were incubated overnight at 4 °C with NOTCH3 primary antibody (diluted 1:300 in PBS). After washing, the cells were treated with a secondary antibody (1:1000 dilution in PBS) for 2 h at room temperature. Finally, nuclei were stained with DAPI for 5 min, and imaged using a fluorescence microscope (Invitrogen M5000 (Thermo Fisher, Waltham, MA, USA)).

### 2.15. Statistical Analysis

All quantitative data are presented as mean ± SD. Statistical comparisons were conducted with one-way ANOVA using GraphPad Prism 9.0, while image analysis was performed with ImageJ software (Fiji Is Just ImageJ). A threshold of *p* < 0.05 was applied to determine statistical significance. Microbiota α-diversity (Sobs, ACE) and β-diversity (Bray–Curtis) were compared using the Kruskal–Wallis test with Benjamini–Hochberg FDR adjustment (adjusted *p* < 0.05). All analyses were conducted in R v4.3.1.

## 3. Results

### 3.1. Dentification of Proteins in BbF

**Figure 1:**
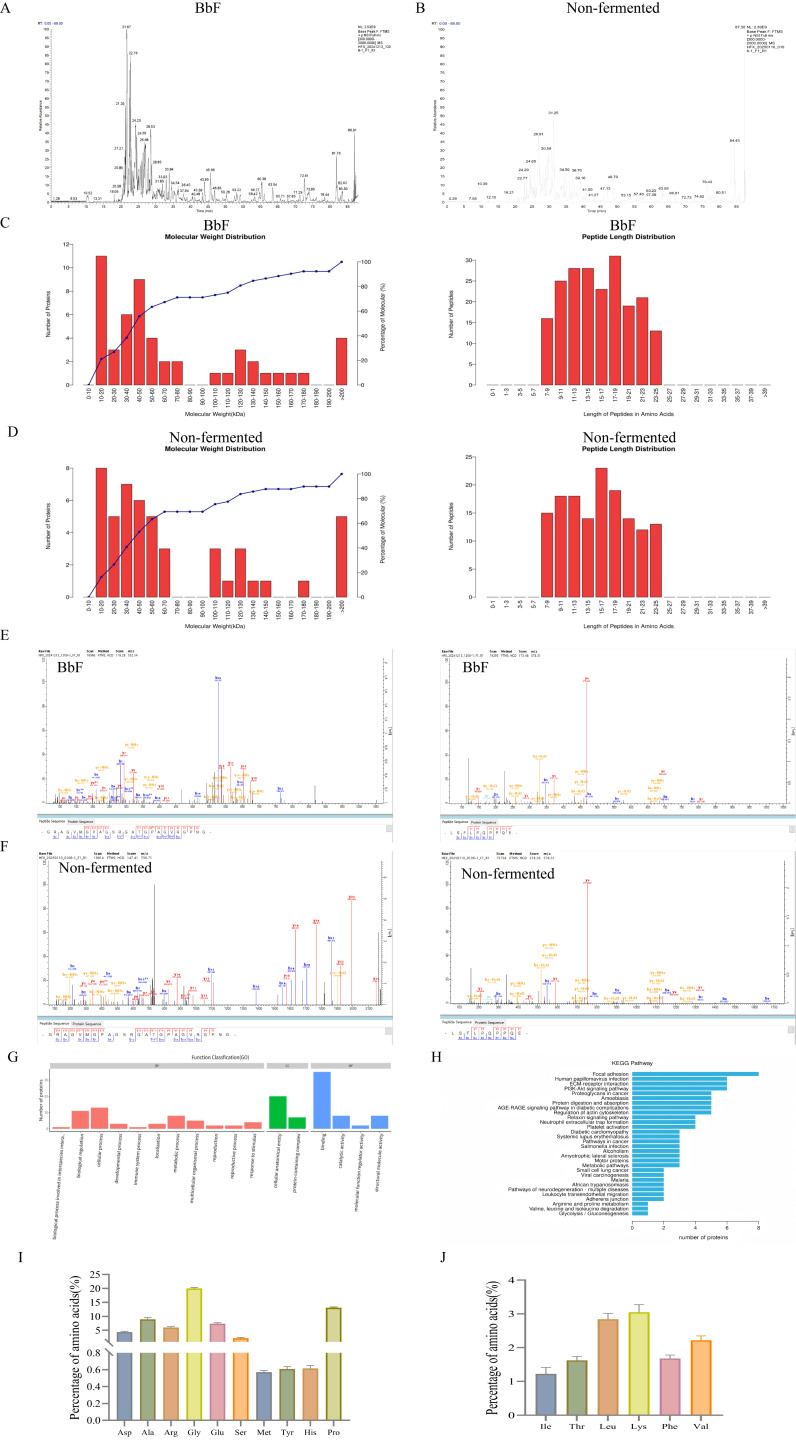
Label-free quantitative proteomics analysis. (**A**) Mass spectrum Basepeak of BbF. (**B**) Mass spectrum Basepeak of non-fermented. (**C**) The length of the BbF peptide and protein molecular weight. (**D**) The length of the non-fermented peptide and protein molecular weight. (**E**) The two most abundant peptides were identified in BbF. (**F**) The two most abundant peptides were identified in non-fermented. (**G**) Functional annotation of differential protein GO. (**H**) Notes on the KEGG pathway of differential protein. (**I**) Amino acid content. (**J**) Essential amino acid content.

Based on proteomic analysis, the fermented product BbF contained 52 proteins and 204 peptides, representing a clear increase from the 49 proteins and 146 peptides identified in the non-fermented water extract. with a majority of the peptides originating from collagen. This expansion in molecular diversity was accompanied by an 8.07% ± 0.26 rise in total protein content. The distribution of peptide lengths, as illustrated in Figure 1C, peptide bonds are more concentrated between 7 and 25. The two peptides exhibiting the most significant signals in both BbF and the aqueous extract were identified as GRAGVMGPAGSRGATGPAGVRGPNG and LSFLPQPPQE, with the peptide intensities in BbF being higher than those in the aqueous extract (Figure 1E,F). Both are peptide fragments from the α1 and α2 chains of type I collagen.

The GO function annotation of differential proteins is illustrated in Figure 1G. A total of 17 biological processes were involved. The molecular function mainly focused on interspecies interaction, biological regulation, cellular processes, developmental processes, immune system processes. A total of three biological processes were found to be associated with the largest number of differential proteins: cellular anatomical entity, structural molecule activity and binding.

Furthermore, KEGG analysis identified 30 relevant pathways associated with digestion and absorption, cytoskeleton regulation, immune system function, and cancer-related signaling (Figure 1H). These pathways are notably closely associated with the autoimmune disease RA.

A total of 15 amino acids were identified in BbF, as illustrated in Figure 1I,J, with glycine being the most abundant (20.337%). The analysis revealed that BbF is not only rich in various non-essential amino acids but also contains a range of amino acids essential for meeting daily human nutritional requirements, supporting physiological metabolic processes, and promoting healthy bone growth and development. Providing a material basis for its subsequent pharmacological effects.

### 3.2. BbF Ameliorates RA Symptoms in an Animal Model

**Figure 2:**
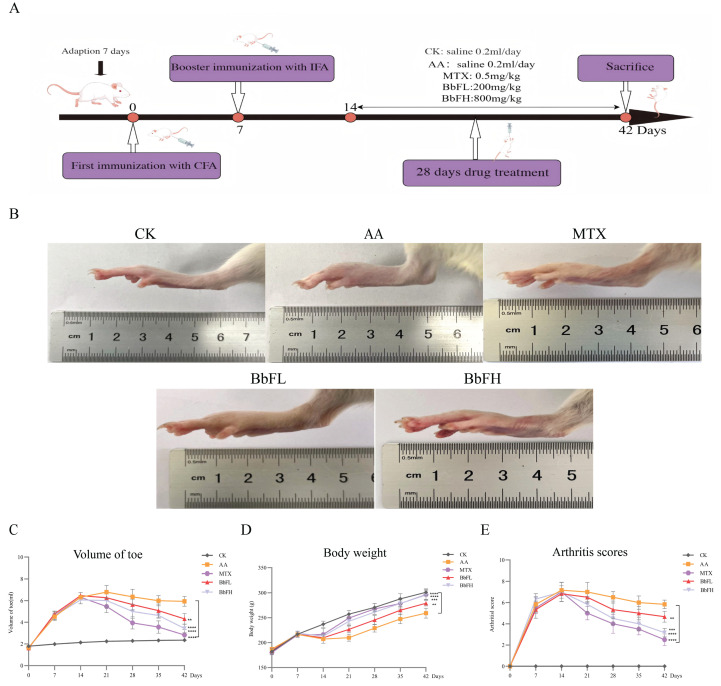
Establishment of rats model of adjuvant RA. (**A**) Timeline of animal experiments. (**B**) Overall observations after treatment for all group. (**C**) Volume of toe. (**D**) Body weight changes. (**E**) Arthritis scores. (** *p* < 0.005 vs. AA. *** *p* < 0.001, **** *p* < 0.0001 vs. AA).

Measurements of toe volume, body weight changes, and joint scores were performed in RA rats (Figure 2C–E). Compared with the AA model group, rats in the BbFH group exhibited a significant reduction in joint swelling and a marked decrease in arthritis scores. The changes in body weight may be attributed to limited mobility caused by swelling and pain, which led to reduced food intake and subsequent weight loss. After treatment with BbFH, swelling was alleviated and body weight was restored—results consistent with the trends observed in the MTX groups. These findings provide strong experimental evidence supporting the potential application of BbF in the treatment of RA.

**Figure 3:**
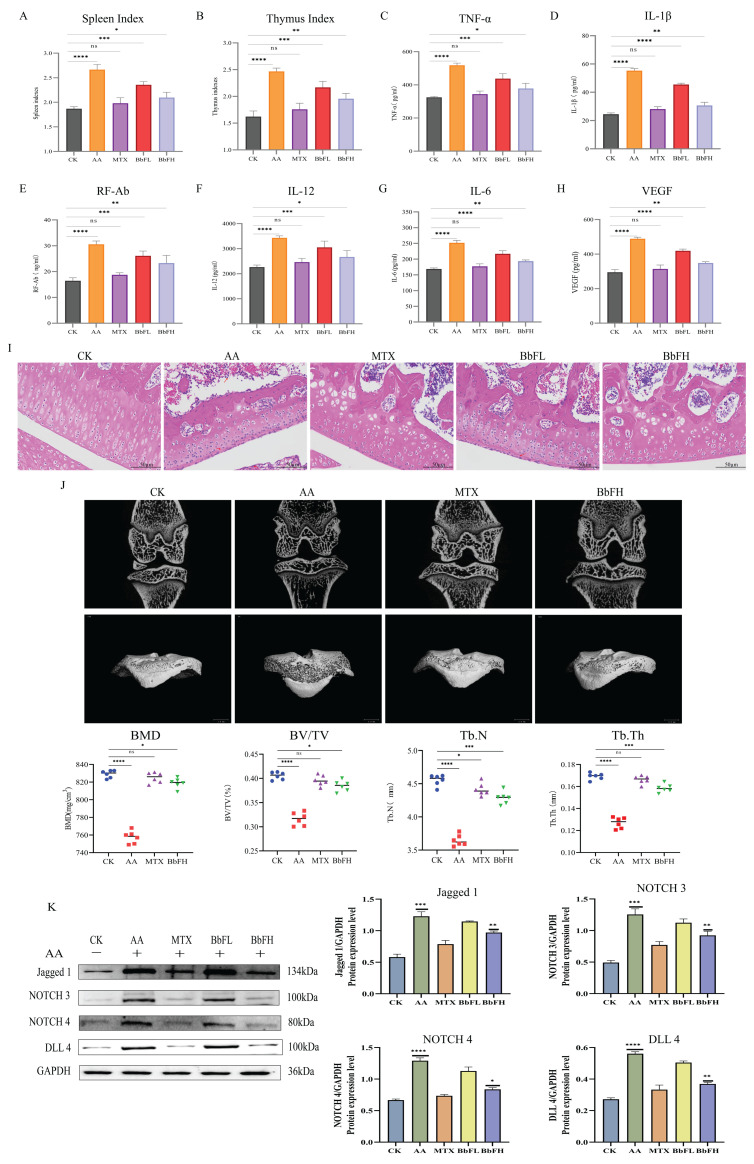
Therapeutic effects of BbF on RA rats. (**A**) Spleen immune index of AA rats in each group. (**B**) Thymic immune index of AA rats in each group. (**C**–**H**) Detection of inflammatory cytokines includes TNF-α, IL-1β, RF-Ab, IL-12, IL-6, VEGF. (**I**) Hematoxylin and eosin (HE) staining. (**J**) 2D micro CT images of rat knee joints. BMD (bone mineral density), Tb.Th (trabecular thickness), Tb.N (number of bone trabeculae) and BV/TV (bone tissue volume/total volume). (**K**) Expressions of related marker proteins and Gray scale analysis of Western blot. (**** *p* < 0.0001, *** *p* < 0.001, ** *p* < 0.01, * *p* < 0.1, ns (not significant) vs. CK).

As shown in the results, the AA group displayed significantly elevated spleen and thymus indices compared to the CK group, reflecting a systemic inflammatory response (Figure 3A,B). Serum levels of multiple inflammatory mediators, including TNF-α, IL-1β, IL-6, IL-12, VEGF, and RF-Ab, were also markedly increased in the AA group (Figure 3C–H). In contrast, all BbF-treated groups showed dose-dependent reductions in both immune organ indices and inflammatory factor levels, aligning with the trends observed in the MTX groups. These results suggest that BbF may ameliorate RA through immunomodulatory mechanisms involving key inflammatory mediators.

To further validate the therapeutic effect of BbF on AA rats, this study also quantified the knee joints of AA rats using micro-CT (Figure 3J). Interestingly, the results showed that knee injuries were significantly lower in the BbFH group treated animals compared to the AA group. This was evidenced by an increase in BV/TV, BMD, Tb.N and Tb.Th.

Histological analysis by HE staining (Figure 3I) demonstrated marked synovial hyperplasia and inflammatory cell infiltration in the AA group relative to the CK group. In the BbFH group, both synovial hyperplasia and inflammatory cell infiltration were markedly alleviated. This effect was consistent with the trends observed in the MTX groups.

In this study, the expression of NOTCH-related proteins NOTCH3, NOTCH4, Delta-like 4 (DLL4) and Jagged1 in RA rat tissues was examined by Western blot (Figure 3K). The results showed that the expression of NOTCH3, NOTCH4, DLL4 and Jagged1 was significantly higher in the AA group compared with the CK group, and the expression of these proteins was significantly lower in the MTX group and BbFH group compared with the AA group, suggesting that BbF significantly inhibited the expression of NOTCH-related proteins. Reduced inflammation level, reduced synovial hyperplasia and alleviated RA.

These findings indicate that the ameliorative effect of BbF on RA is closely associated with the NOTCH signaling pathway, providing new insights and strategies for the treatment of this disease.

### 3.3. Transcriptomic Changes in Rat

#### 3.3.1. Transcriptome Sequencing and DEGs Identification

**Figure 4:**
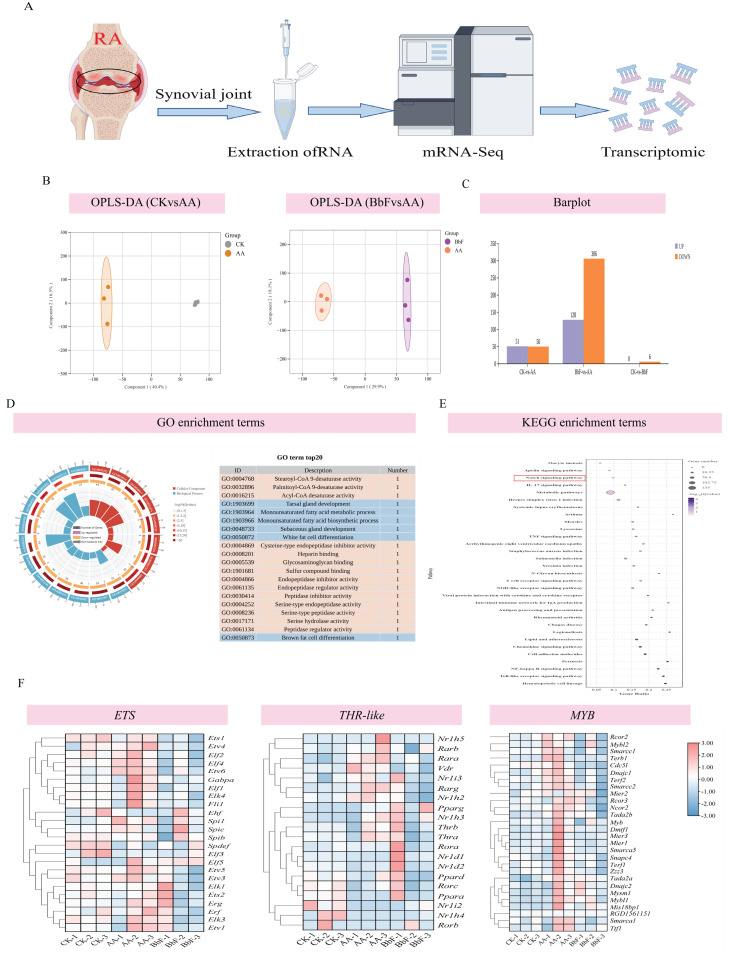
Transcriptomics analysis (n = 3). (**A**) Transcriptome sequencing. (**B**,**C**) Partial least squares discriminant analysis (OPLS-DA). (**D**) GO enrichment analysis. (**E**) KEGG pathway analysis. The red box highlights the Notch signaling pathway. (**F**) ETS, MYB, and Thr-like gene families.

Sequencing of RA synovial tissues yielded 55,714,152 clean reads from 9 samples, resulting in 8.277 Gb of clean base data, with more than 98% of Q20 and 94% of Q30 bases, and close to 50% GC. 23,140 Unigenes were detected, and partial least squares discriminant analysis (OPLS-DA) showed that BbF is effective in the treatment of RA. A total of 23,140 Unigenes were identified. Partial least squares-discriminant analysis (OPLS-DA) revealed clear separation between experimental groups, indicating a distinct transcriptional response following BbF treatment (Figure 4B,C). Using the thresholds |log_2_FC| ≥ 1 and FDR < 0.05, 1504 differentially expressed genes (DEGs) were detected between the CK and AA groups, including 1466 up-regulated and 38 down-regulated genes. Furthermore, 6863 DEGs were identified between the AA and BbF groups, with 982 up-regulated and 5881 down-regulated (Figure 4C).

#### 3.3.2. Analysis of GO and KEGG Pathways in DEGs

The DEGs identified in this study were annotated using the public database Metascape. GO enrichment analysis revealed that DEGs were significantly enriched in biological processes such as extracellular region, extracellular space, cell periphery, inflammatory response, humoral immune response, and heparin binding. After BbF treatment, regulation of blood circulation, regulation of blood pressure, cell differentiation, regulation of signaling, and endocrine process were altered (Figure 4D). Additionally, KEGG annotation results showed that DEGs were involved in various biological pathways, including environmental information processing, genetic information processing, metabolism, and organismal systems. Key pathways affected by DEGs included cytochrome NOTCH signaling pathway, IL-17 signaling pathway, Herpes simplex virus 1 infection, Systemic lupus erythematosus, Asthma, Measles, Lysosome, TNF signaling pathway, T cell receptor signaling pathway, NOD-like receptor signaling pathway (Figure 4E).

#### 3.3.3. Effects of ETS, MYB, Thr-like Gene Families on Rats

Typical symptoms of RA include joint swelling, morning stiffness and symmetrical joint involvement. Transcriptomic studies suggest that the ETS, MYB, and Thr-like gene families may play key roles in the pathology of RA (Figure 4F). The ETS family acts as transcriptional regulators involved in the activation of inflammatory signaling pathways (e.g., the IL-17 and TNF pathways), which may exacerbate synovial inflammation by regulating chemotaxis and activation of immune cells (e.g., T cells and macrophages). Abnormal expression of MYB family genes, which are commonly associated with cell differentiation and proliferation, may lead to synovial fibroblast proliferation and joint destruction. Meanwhile, the Thr-like family may regulate aberrant metabolic and immune homeostasis in RA by modulating cell signaling pathways such as the PI3K-Akt or AMPK pathways. Experimental data suggest that ETS and MYB family members are significantly upregulated in RA models, leading to excessive release of inflammatory factors (e.g., IL-6, TNF-α) and dysregulation of autoimmune responses. Conversely, aberrant expression of Thr-like genes may disrupt the balance between apoptosis and repair, further promoting joint damage. After BbF treatment, the expression levels of these genes returned to baseline, suggesting that their dynamic regulation is closely associated with RA pathologic remission. In addition, KEGG pathway analysis highlighted that these gene families may synergistically affect RA-associated immune dysregulation and tissue remodeling through chemokine signaling, NOTCH pathway, and metabolic processes, providing potential molecular targets for precision therapy.

### 3.4. BbF Restores Gut Microbiota Homeostasis and SCFAs Production

**Figure 5:**
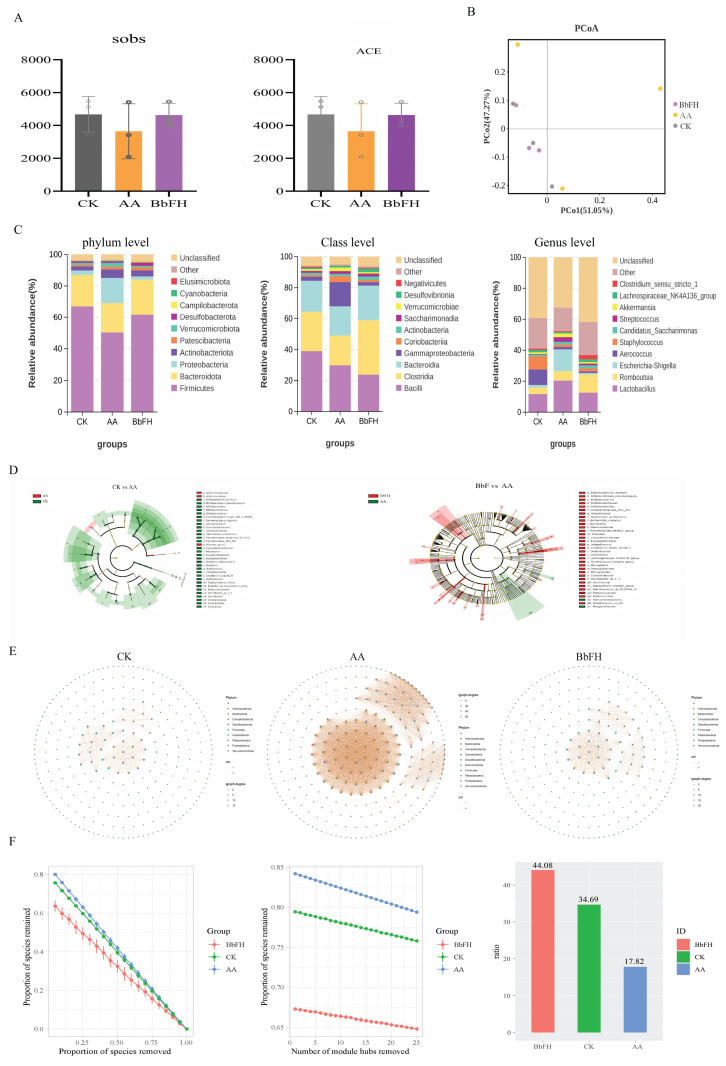
Gut microbiota diversity and composition. (**A**) α-diversity. (**B**) β-diversity. (**C**) phylum, order, and genus level colony analysis. (**D**) LEfSe analysis. (**E**,**F**) Covariate network analysis.

As illustrated in Figure 5A, the Sobs index and ACE index of the BbFH group exhibited significantly higher values in comparison to those of the AA group. The findings of this study suggest that BbFH treatment enhances both the richness and diversity of the fecal bacterial community. Furthermore, an analytical investigation was undertaken to ascertain the similarities and differences between the three samples. As demonstrated in Figure 5B, a clear separation was evident between the AA group and the CK group, while no significant separation was observed for the BbFH group.

To further investigate the compositional changes in the intestinal microbiota of RA rats induced by BbF, the species composition at the phylum, class, and genus levels in the fecal samples of the CK, AA, and BbF groups was explored, as shown in Figure 5C.

At the phylum level, the predominant bacterial communities detected across all three groups were *Firmicutes* and *Bacteroidota*. Compared with the CK group, the abundance of *Firmicutes* was significantly decreased in the AA group, while the abundance of *Proteobacteria* was markedly increased. Studies have indicated that depletion of *Firmicutes* in RA patients may lead to reduced immunity [25,26]. *Proteobacteria*, as representatives of Gram-negative bacteria, may indirectly promote RA pathogenesis through immune response activation [27]. It is noteworthy that BbFH treatment reversed this phenomenon, restoring the microbial composition to a state closer to that of the CK group.

At the class level, the top five bacterial classes identified were *Bacilli, Clostridia, Bacteroidia, Gammaproteobacteria,* and *Coriobacteriia,* with *Bacilli* being the most abundant. In the AA group, the abundances of *Clostridia* and *Bacteroidia* decreased, while that of *Gammaproteobacteria* increased significantly. This shift may contribute to the progression of RA. *Clostridia* and *Bacteroidia* are known to play crucial roles in the production of SCFAs, whereas *Gammaproteobacteria* promote the secretion of inflammatory cytokines and play a key role in RA pathogenesis [28]. Following BbFH intervention, the abundances of Clostridia and *Bacteroidia* increased, while that of *Gammaproteobacteria* decreased, thereby alleviating inflammation, ameliorating RA symptoms, and restoring gut microbiota balance.

At the genus level, compared with the CK group, the AA group exhibited an abnormal increase in the abundance of *Escherichia-Shigella*. Research has shown that this genus can activate the Toll-like receptor 4 (TLR4) signaling pathway, promoting the secretion of pro-inflammatory cytokines such as TNF-α and IL-6, and exacerbating synovitis [29]. After BbFH intervention, the proliferation of *Escherichia-Shigella* was significantly inhibited. These results demonstrate that BbFH helps restore and maintain gut microbiota homeostasis in RA.

The genera identified through LEfSe analysis to detect significant differences between the AA group and the BbFH intervention group are presented in Figure 5D. In contrast to the AA group, the relative abundances of *Bifidobacterium*, *Rikenella*, *Barnesiella*, *Eubacterium siraeum group*, *Oscillibacter* and *Monoglobus* were significantly altered in the BbFH administration group (LDA > 2, *p* < 0.05). The present findings suggest that BbFH intervention may influence the immunopathological process of RA by modulating the abundance of these bacterial genera and enhancing the metabolic function of the gut microbiota, such as SCFA production.

The covariance network plot reveals the abundance of RA intestinal flora, as shown in Figure 5E. The three groups showed differences, with the AA group having a more complex network abundance compared to the CK group, which was effectively reversed by BbFH administration, making it more stable and almost close to the CK group. As shown in Figure 5F, the BbFH group significantly increased the proportion of negative correlation, bringing it closer to CK.

**Figure 6:**
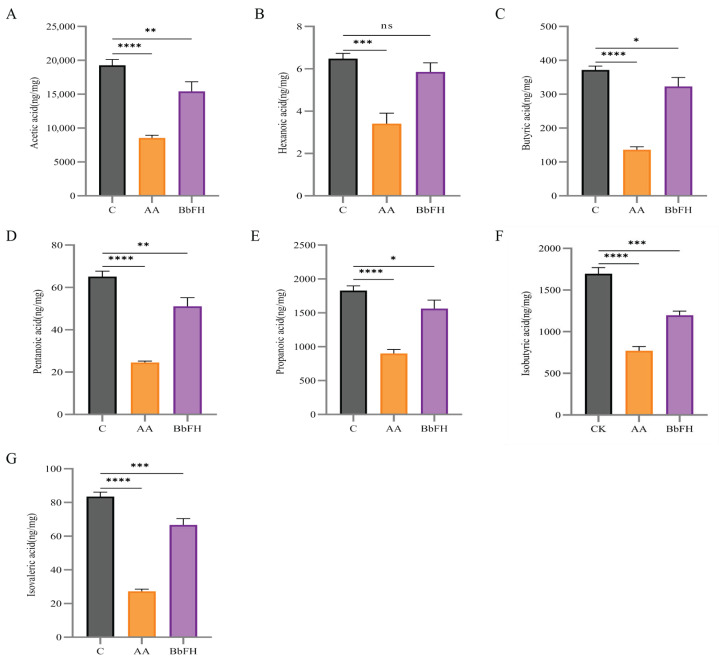
Effects on SCFA levels in feces of RA rats. (**A**) Acetic acid content. (**B**) Hexanoic acid content. (**C**) Butyric acid content. (**D**) Pentanoic acid content. (**E**) Propionic acid content. (**F**) Isobutyric acid content. (**G**) Isovaleric acid content. (**** *p* < 0.0001, *** *p* < 0.001, ** *p* < 0.01, * *p* < 0.1, ns (not significant) vs. CK).

The fecal levels of seven SCFAs—including Acetic acid, Hexanoic acid, Butyric acid, Pentanoic acid, Propionic acid, Isobutyric acid, Isovaleric acid—were quantified in RA model rats. As illustrated in Figure 6A–G, SCFA concentrations were markedly reduced in the AA group relative to the CK group. In contrast, BbFH treatment significantly increased the levels of caproic, butyric, and propionic acids, restoring them to near-normal levels comparable to those in the CK group. These findings suggest that BbF not only restructures the gut microbiota but also enhances SCFA production, potentially reinforcing the intestinal mucus barrier in RA rats.

### 3.5. Evaluation of the Cytotoxic Effects of BbF on RAW264.7 Cells

**Figure 7:**
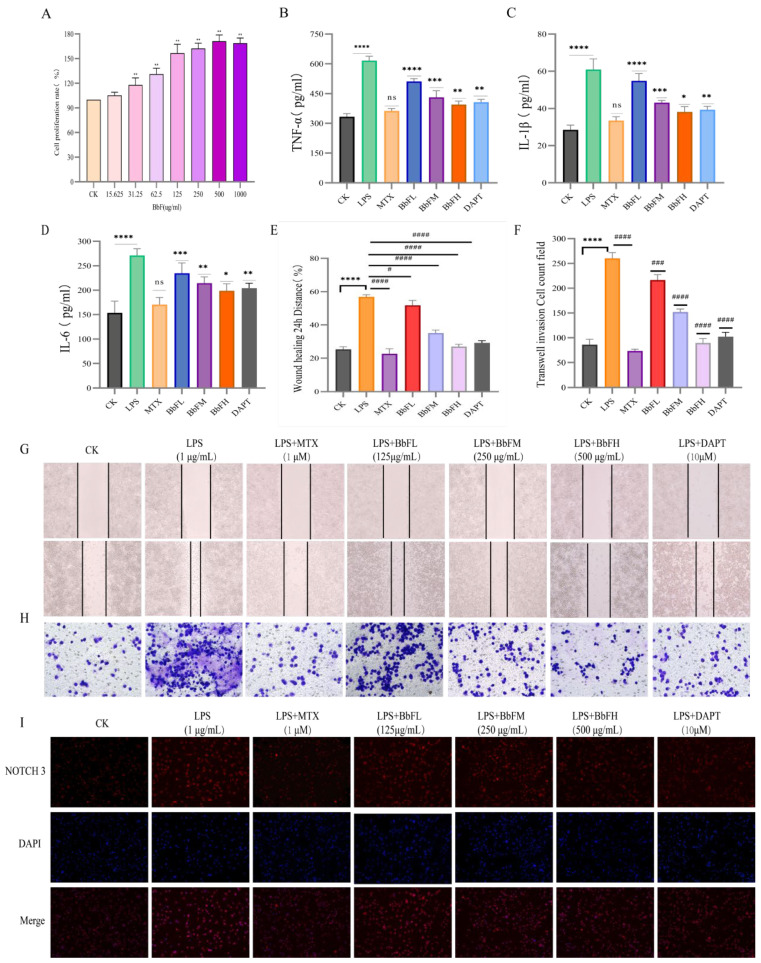
Influence of BbF on the Anti-inflammatory Activity, Invasion and Migration of RA-RAW264.7 Cells. (**A**) Effect of BbF on the viability of RAW264.7 cells (** *p* < 0.01). (**B**–**D**) Inflammatory cytokines measured in cell culture supernatant include TNF-α, IL-1β, and IL-6. (**E**–**H**) RA-RAW264.7 cell migration and invasion. (**I**) Immunofluorescence detection of NOTCH 3 in RA-RAW264.7 cells. (**** *p* < 0.0001, *** *p* < 0.001, ** *p* < 0.01, * *p* < 0.1, ns (not significant) vs. CK; #### *p* < 0.0001, ### *p* < 0.001, # *p* < 0.1, ns (not significant) vs. LPS).

For the purpose of assessing the effect of BbF on the toxicity of RAW264.7. The concentration range was set at 15.625~1000 μg/mL, in Figure 7A shows that BbF does not produce any adverse effects on RAW264.7 cells when the concentration of BbF is lower than 1000 μg/mL, and the activity of RAW264.7 cells was dose-dependent with a concentration range of 15.625~1000 μg/mL. Therefore, concentrations of 125, 250, and 500 μg/mL were selected for subsequent experiments.

Cell migration and invasion assays revealed that the LPS group displayed significantly greater migration compared to the CK group, whereas the BbFH, MTX, and DAPT groups all exhibited markedly suppressed migration. Notably, BbFH showed a stronger inhibitory effect than BbFL (Figure 7E,G). A similar trend was observed in invasion assays, with the LPS group showing a pronounced increase in invaded cells relative to CK, while all treatment groups effectively inhibited invasion. These results demonstrate that BbF significantly suppresses both migration and invasion of RA-RAW264.7 cells (Figure 7F,H).

To evaluate the anti-inflammatory activity of BbF, an in vitro inflammatory model was established by stimulating RAW264.7 cells with LPS (Figure 7B–D). ELISA analysis of the culture supernatant showed that BbF treatment significantly decreased the secretion of key pro-inflammatory cytokines, including TNF-α, IL-1β, and IL-6, compared to the LPS group. These findings suggest that BbF attenuates inflammation by suppressing the release of inflammatory mediators.

Immunofluorescence experiments further showed that compared with the control group, NOTCH3 fluorescence intensity was significantly enhanced in the LPS group, and NOTCH3 gradually ablated after BbFH administration compared with the LPS group, which was able to inhibit LPS-induced expression of NOTCH3 in RA-RAW264.7 cells, suggesting that the mechanism of RA-RAW264.7 cells in NOTCH deserves further research (Figure 7I).

### 3.6. Inhibition of the NOTCH Signaling Pathway Can Alleviate RA

The results demonstrate that the NOTCH signaling pathway plays a critical role in the treatment of rheumatoid arthritis (RA). Activation of the NOTCH signaling pathway promotes the proliferation and differentiation of synovial fibroblasts, regulates macrophages, enhances the secretion of pro-inflammatory cytokines, and exacerbates inflammation. Based on these findings, it was hypothesized that probiotic-fermented deer bone aqueous extract (BbF) could alleviate RA symptoms by inhibiting the NOTCH signaling pathway, similar to the action of DAPT (a γ-secretase inhibitor).

**Figure 8:**
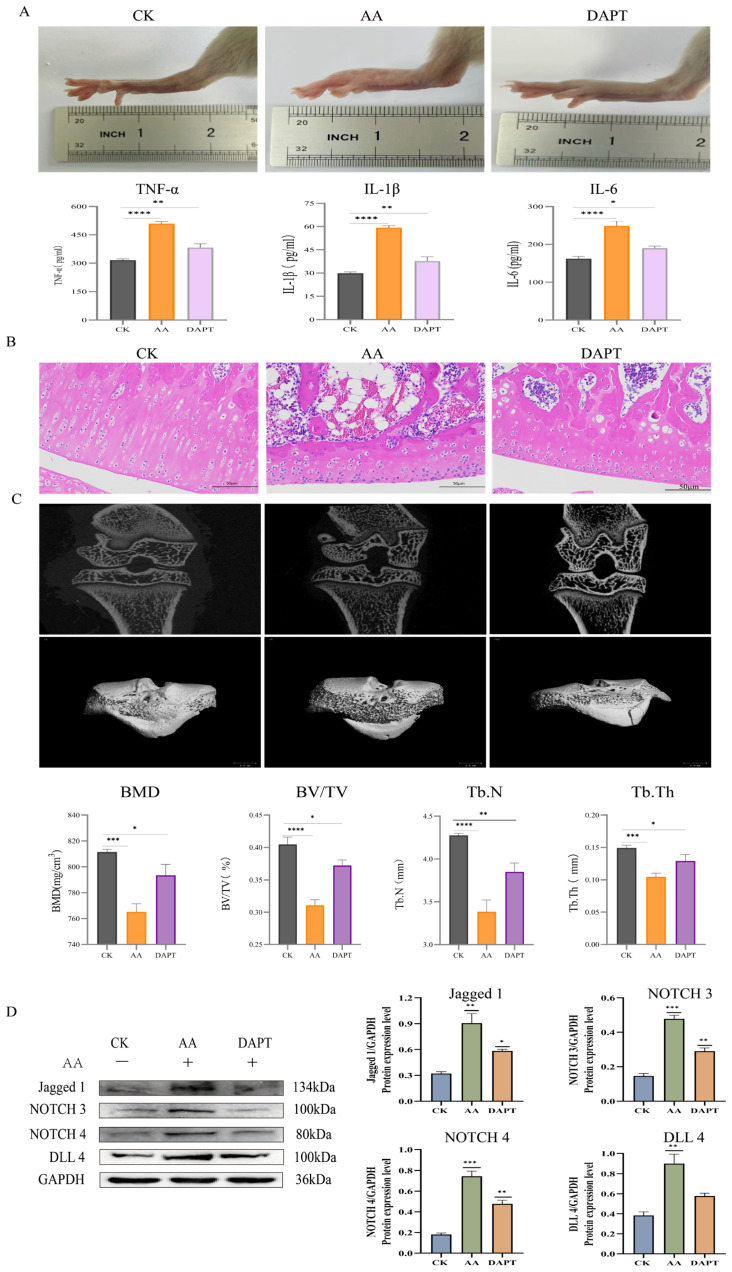
DAPT inhibits the activation of the NOTCH signaling pathway. (**A**) DAPT reduces the severity of swelling and inflammatory factor assays in RA. (**B**) Hematoxylin and Eosin Staining. (**C**) DAPT alleviates bone erosion in RA. (**D**) Expressions of related marker proteins and Gray scale analysis of Western blot. (**** *p* < 0.0001, *** *p* < 0.001, ** *p* < 0.01, * *p* < 0.1, ns (not significant) vs. CK).

The results, as shown in the phenotype images and inflammatory factor assays (Figure 8A), indicate that DAPT effectively suppressed inflammation in RA rats. Compared to the RA model group, DAPT treatment significantly reduced paw swelling, with the DAPT-treated group showing a 65% reduction (*p* < 0.01) and the BbF-treated group showing a 60% reduction (*p* < 0.01). Additionally, histological analysis using Hematoxylin and Eosin (HE) staining (Figure 8B) and micro-CT imaging (Figure 8C) revealed significant alleviation of synovial hyperplasia, reduced inflammatory cell infiltration, and an overall reduction in bone erosion. These results were consistent with the effects observed in the BbF-treated group, further supporting the hypothesis that BbF alleviates RA by inhibiting the NOTCH pathway.

Western blot analysis (Figure 8D) was performed to examine the expression of key NOTCH signaling proteins, including NOTCH3, NOTCH4, DLL4, and Jagged1. The results revealed that both DAPT and BbF treatments significantly reduced the expression of these proteins, confirming that BbF effectively inhibits the NOTCH signaling pathway. The consistent inhibitory effects of both DAPT and BbF on NOTCH pathway proteins provide strong evidence for BbF’s role in alleviating RA through the inhibition of the NOTCH signaling pathway.

## 4. Discussion

This research investigates the regulatory role of BbF in modulating immune responses and maintaining intestinal microbiota homeostasis within a rat model of RA. The findings are expected to not only enhance our comprehension of BbF’s active components but also to introduce new paradigms for RA therapy.

As a chronic, systemic, and symmetrical autoimmune disorder, RA affects a significant portion of the global population and is clinically defined by joint inflammation and synovial proliferation [30]. Although current treatment options predominantly consist of synthetic drugs, they are often limited by serious side effects, which remains a major concern for patients.

Deer bone is renowned as a natural biological architect of the skeletal framework, has increasingly been shown to possess bioactive components with anti-inflammatory and anti-osteoporosis effects [31]. This study utilizes *Bifidobacterium breve* to ferment the deer bone extract to obtain the BbF product. The choice of *Bifidobacterium breve* as the fermenting organism is biologically coherent. *Bifidobacterium breve* is a well-characterized probiotic with documented anti-inflammatory activity and barrier-supporting effects in intestinal models, and it has been associated with improved outcomes in immune-mediated conditions [32,33]. Fermenting a protein-rich deer bone extract with *Bifidobacterium breve* likely generates a distinct spectrum of low-molecular-weight peptides and amino acid derivatives with bioactivity. Our preliminary experiments demonstrated that *Bifidobacterium breve* exhibits robust growth and pronounced protease activity when cultured in deer bone water extract, efficiently hydrolyzing deer bone-derived proteins to release bioactive peptides. This enzymatic biotransformation process directly accounts for the higher protein diversity and greater peptide abundance observed in BbF compared to the unfermented deer bone water extract. This is crucial for maintaining the mechanical properties of articular cartilage and the stability of the extracellular matrix. These findings highlight the importance of microbial fermentation in enhancing the functional properties of BbF.

The transcriptomic analysis of the CK, AA, and BbF groups revealed that the NOTCH signaling pathway was significantly enriched in the differential gene enrichment analysis, suggesting its potential role in the amelioration of RA by BbF. The NOTCH protein, as a critical transmembrane receptor involved in cell growth and development, plays a key role in the differentiation of immune cells such as T and B cells and is closely associated with immune responses [34]. In RA patients, the expression of NOTCH ligands and receptors (e.g., JAG1-2, DLL1-4, NOTCH1-3) is significantly elevated in synovial tissues, indicating their aberrant activation in RA [35]. Inhibiting the NOTCH signaling pathway can reduce the release of pro-inflammatory cytokines, thereby alleviating RA symptoms and providing organ-protective effects [36]. Currently, γ-secretase inhibitors such as DAPT and LY411575 are commonly used to inhibit the NOTCH pathway, but they exhibit gastrointestinal side effects [37]. Therefore, the development of natural, low-toxicity inhibitors is of great importance. To validate the hypothesis that BbF inhibits the NOTCH pathway to ameliorate RA, we introduced the NOTCH-specific inhibitor DAPT as a positive control to further investigate the mechanisms of BbF in both in vivo and in vitro experiments.

In vivo and in vitro results demonstrated that BbF significantly improved bone density, suppressed synovial hyperplasia, reduced key pro-inflammatory cytokines (TNF-α, IL-1β, IL-6), and inhibited macrophage migration and invasion. This coordinated pattern of regulation aligned with the effects observed in the DAPT group, further supporting the central role of the NOTCH pathway in the therapeutic process. Notably, BbF exhibited superior safety profile compared to MTX. During experimentation, diarrhea observed in select MTX-treated animals was likely treatment-related [38], whereas no such adverse effects were detected in the BbF group. Molecular analyses through Western blot and immunofluorescence confirmed BbF effectively suppressed expression of key NOTCH pathway proteins (NOTCH3, NOTCH4, Jagged1, DLL4), this observation is consistent with findings reported by Sha Ma et al. [39]. These findings provide direct evidence that BbF exerts anti-RA effects through NOTCH signaling pathway inhibition, thereby validating our initial hypothesis.

In the RA model, pro-inflammatory factors such as TNF-α, IL-6, and IL-1β, which are significantly elevated in the joints of rats, can enter the systemic circulation and subsequently act directly on intestinal epithelial immune cells, thereby inducing gut microbiota dysbiosis [40]. While a connection between RA and gut microbiota is recognized, the precise mechanisms underlying its pathological influence are still being unraveled. In our experimental model, we observed a substantial decline in the abundance and diversity of the intestinal flora in RA rat. The depletion of *Firmicutes* and *Bacteroidota* reduces SCFA production, triggering inflammatory bowel disease [41]. Studies confirm that inflammatory bowel disease serves as a significant inducing factor for rheumatoid arthritis by perpetuating systemic inflammation and immune dysregulation, thereby establishing a pathogenic gut-joint axis pathway [42]. The significant enrichment of *Proteobacteria* (a key microbial marker of gut dysbiosis), along with *Gammaproteobacteria* and *Escherichia-Shigella*, in our RA model further disrupts intestinal homeostasis and exacerbates inflammatory responses. This indicates that the onset of RA induces a state of enteric bacteriosis [43]. Particularly important, the present study observed the positive effects of BbF on the intestinal flora of RA rats and BbF significantly altered the diversity and structure of the intestinal flora of RA rats, increasing the abundance of beneficial bacteria such as *Firmicutes*, *Bacteroidota*, and *Clostridia*, while decreasing the abundance of harmful bacteria *Proteobacteria*, *Gammaproteobacteria* and *Escherichia-Shigella*, among others. These changes not only help to restore the homeostasis of the intestinal flora, but also can enhance the intestinal barrier function by affecting the production of SCFAs. SCFAs such as acetate, propionate, and butyrate can promote Treg differentiation, temper Th17 responses, and enhance epithelial integrity [44], among which butyrate serves as a crucial energy source for colonocytes [45]. In the context of autoimmune diseases, butyrate demonstrates potent anti-inflammatory properties and plays a vital role in maintaining intestinal epithelial health and restoring gut barrier function [46]. Our findings reveal that BbF significantly enhanced the levels of SCFAs, particularly butyrate, indicating its capacity to repair the intestinal barrier. These results underscore the important impact of BbF on gut regulation in rheumatoid arthritis. Although BbF exerted clear modulatory effects on gut microbiota composition, these results were derived from a small sample size (n = 3 per group), which may limit statistical power. Future studies with larger cohorts and metagenomic validation are warranted to confirm these community-level shifts.

In summary, this study demonstrates that BbF ameliorates RA in an animal model BbF modified the gut microbiota composition and exerted anti-inflammatory effects associated with immune cell regulation.

## 5. Conclusions

This study reveals for the first time that BbF alleviates RA by inhibiting the NOTCH signaling pathway and modulating the gut microbiota. BbF effectively rebalances aberrant immune responses and restores gut homeostasis in RA models, leading to significant reductions in inflammatory joint damage. As a natural product with low toxicity and high safety, BbF demonstrates a unique immunomodulatory and anti-inflammatory potential, highlighting a novel functional food-based strategy for RA management. Future translational research should evaluate the bioavailability, long-term safety, and efficacy of BbF in human RA, potentially as a functional food-based adjunct to existing therapy.

## Data Availability

The original contributions presented in the study are included in the article. Further inquiries can be directed to the corresponding authors.

## Abbreviations

The following abbreviations are used in this manuscript:

RA	Rheumatoid arthritis
BbF	*Bifidobacterium breve*-fermented deer bone aqueous extract
CK	Control group
AA	Adjuvant-induced arthritis (model) group
MTX	Methotrexate
DAPT	A γ-secretase inhibitor
BbFH	High-dose *Bifidobacterium breve*-fermented deer bone water extract (animal: 800 mg/kg; cell: 500 μg/mL)
BbFL	Low-dose *Bifidobacterium breve*-fermented deer bone water extract (animal: 200 mg/kg; cell: 125 μg/mL)
BbFM	Medium-dose *Bifidobacterium breve*-fermented deer bone water extract (cell: 250 μg/mL)
SCFA	Short-chain fatty acid
TNF-α	Tumor Necrosis Factor-alpha
IL-1β	Interleukin-1 beta
IL-6	Interleukin-6
IL-12	Interleukin-12
VEGF	Vascular Endothelial Growth Factor
RF-Ab	Rheumatoid Factor Antibody
BV/TV	Bone Volume
BMD	Bone Mineral Density
Tb.N	Trabecular Number
Tb.Th	Trabecular Thickness
