# Correction: Ma et al. Effects of Probiotic-Fermented Deer Bone Water Extract on Immune Regulation and Gut Microbiota in Rheumatoid Arthritis via the NOTCH Signaling Pathway. *Foods* 2025, *14*, 3802

**DOI:** 10.3390/foods15020327

**Published:** 2026-01-16

**Authors:** Junxia Ma, Yingshan Jiang, Yue Teng, Ting Ren, Yanchao Xing, Aoyun Li, Zhongmei He, Weijia Chen, Ying Zong, Rui Du

**Affiliations:** 1 College of Chinese Medicinal Material, Jilin Agricultural University, Changchun 130118, China; jxiama@163.com (J.M.);; 2 Laboratory of Production and Product Application of Sika Deer of Jilin Province, Jilin Agricultural University, Changchun 130118, China; 3 College of Agriculture, Yanbian University, Yanji 133002, China

In the original publication [1], there were two mistakes in Figures 7 and 8 as published. During the manuscript preparation process, a copy–paste error occurred in the image layout, resulting in a duplication issue. Specifically, the 0 h photograph of the CK group in Figure 7G overlaps with that of the LPS+MTX group at the same time point. Additionally, the 2D cross-sectional image of the DAPT group in Figure 8C (Micro-CT image) is identical to that of the BbFH group in Figure 3J (Micro-CT image). The corrected Figure 7 and Figure 8 appear below.

The authors state that the scientific conclusions are unaffected. This correction was approved by the Academic Editor. The original publication has also been updated.

## Figures and Tables

**Figure 7:**
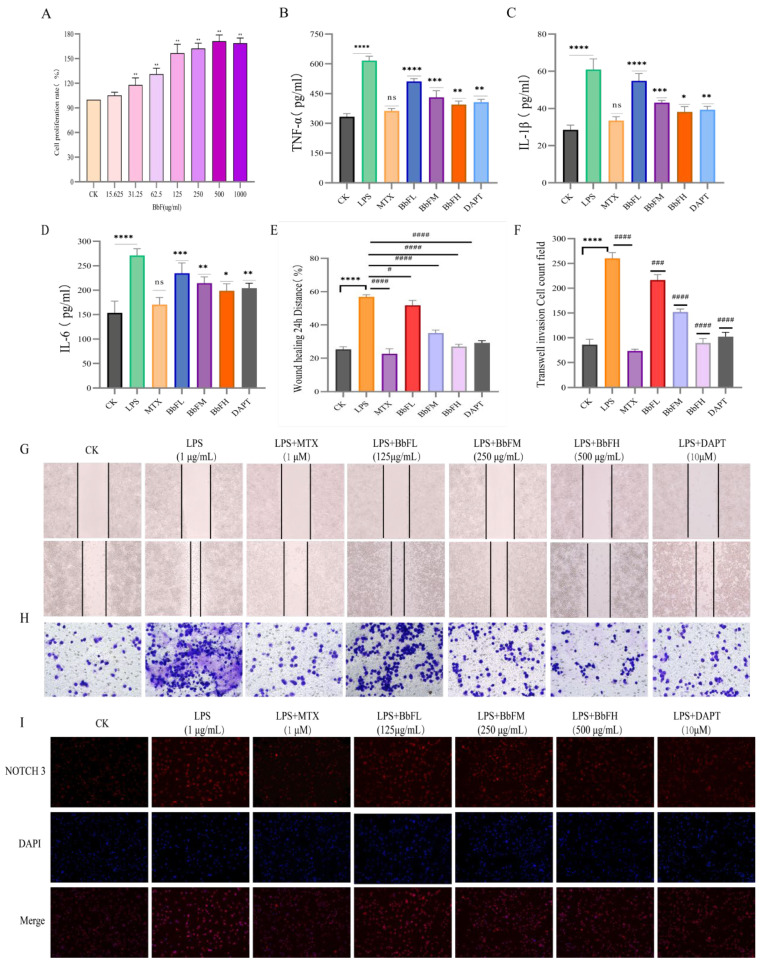
Influence of BbF on the Anti-inflammatory Activity, Invasion and Migration of RA-RAW264.7 Cells. (**A**) Effect of BbF on the viability of RAW264.7 cells (** *p* < 0.01). (**B**–**D**) Inflammatory cytokines measured in cell culture supernatant include TNF-α, IL-1β, and IL-6. (**E**–**H**) RA-RAW264.7 cell migration and invasion. (**I**) Immunofluorescence detection of NOTCH 3 in RA-RAW264.7 cells. (**** *p* < 0.0001, *** *p* < 0.001, ** *p* < 0.01, * *p* < 0.1, ns (not significant) vs. CK; #### *p* < 0.0001, ### *p* < 0.001, # *p* < 0.1, ns (not significant) vs. LPS).

**Figure 8:**
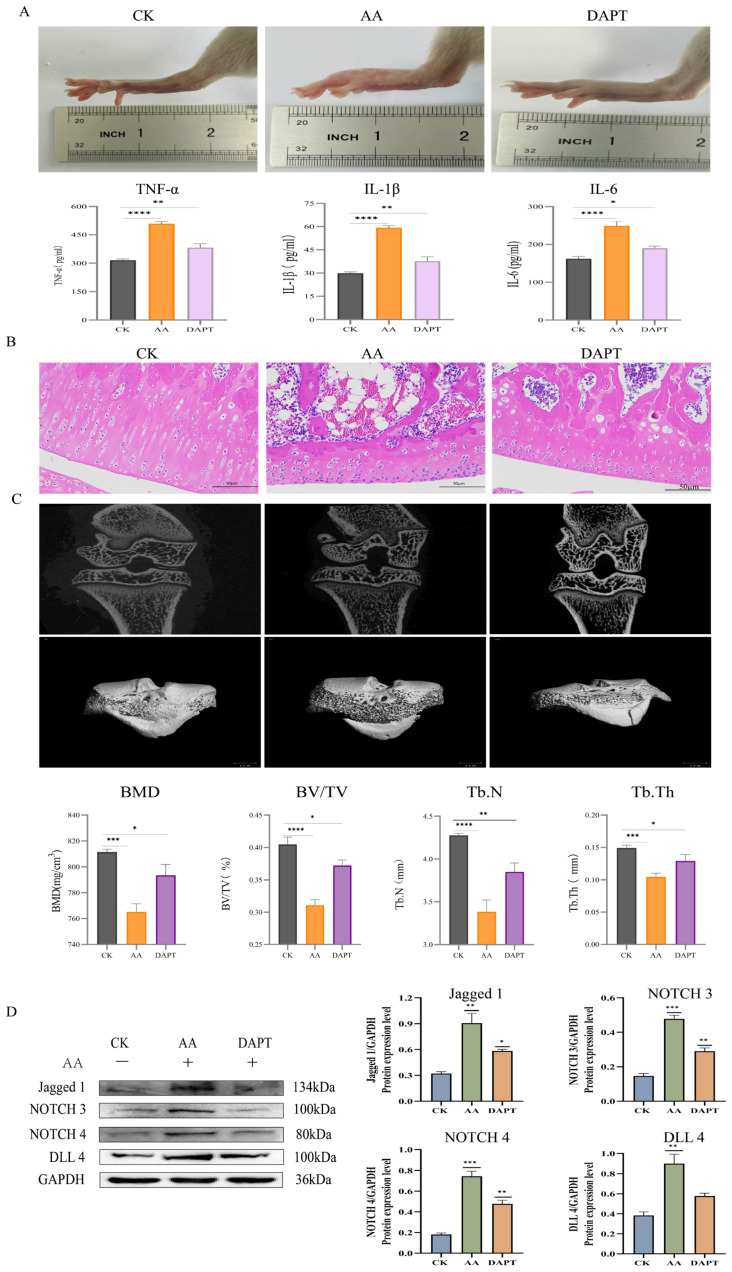
DAPT inhibits the activation of the NOTCH signaling pathway. (**A**) DAPT reduces the severity of swelling and inflammatory factor assays in RA. (**B**) Hematoxylin and Eosin Staining. (**C**) DAPT alleviates bone erosion in RA. (**D**) Expressions of related marker proteins and Gray scale analysis of Western blot. (**** *p* < 0.0001, *** *p* < 0.001, ** *p* < 0.01, * *p* < 0.1, ns (not significant) vs. CK).
